# Multi-Omics Research Strategies for Psoriasis and Atopic Dermatitis

**DOI:** 10.3390/ijms24098018

**Published:** 2023-04-28

**Authors:** Youming Guo, Lingling Luo, Jing Zhu, Chengrang Li

**Affiliations:** Jiangsu Key Laboratory of Molecular Biology for Skin Diseases and STIs, Nanjing 210042, China; 1610122619@pku.edu.cn (Y.G.);

**Keywords:** multi-omics, psoriasis, atopic dermatitis, 3D genomics, transcriptomics, proteomics, metabolomics, microbiomics

## Abstract

Psoriasis and atopic dermatitis (AD) are multifactorial and heterogeneous inflammatory skin diseases, while years of research have yielded no cure, and the costs associated with caring for people suffering from psoriasis and AD are a huge burden on society. Integrating several omics datasets will enable coordinate-based simultaneous analysis of hundreds of genes, RNAs, chromatins, proteins, and metabolites in particular cells, revealing networks of links between various molecular levels. In this review, we discuss the latest developments in the fields of genomes, transcriptomics, proteomics, and metabolomics and discuss how they were used to identify biomarkers and understand the main pathogenic mechanisms underlying these diseases. Finally, we outline strategies for achieving multi-omics integration and how integrative omics and systems biology can advance our knowledge of, and ability to treat, psoriasis and AD.

## 1. Introduction

Psoriasis is a chronic inflammatory disease characterized by immune-mediated inflammation and abnormal keratinocyte differentiation, occurring worldwide and affecting over 60 million adults and children [1]. Equally common is atopic dermatitis (AD), featured by persistent itching, whose prevalence has been reported up to 20% among children and 10% among adults [2]. Psoriasis and AD both have a complicated etiology that involves cytokine and immune cell imbalances. Environmental variables, genetic factors in skin barriers, dysbiosis of skin-resident microbiomes, and immune system deficiencies are only a few of the possible causes of psoriasis and AD progression that can be unraveled by a combination of research from several angles [3,4]. The omics research opens a window to target gene discovery and drug repurposing in psoriasis and AD by providing multidimensional perspectives on the disease.

The central dogma, which states the transfer of information from DNA to DNA/RNA or from nucleic acid to protein [5], is the keystone of molecular biology, leading to different omics approaches widely used today, including genomics, transcriptomics, and proteomics (Figure in Section 3.1) As the Human Genome Project was completed in 2001, the first omics approach, genomics, was driven by technological developments that allowed for the affordable study of whole genomes instead of individual variants or single genes. Subsequently, based on hybridization of cDNA to arrays of oligonucleotide capture probes, the mRNA expression array was developed and continuously improved to the extent that all protein-coding transcripts could be quantified. With the rapid development of high-throughput RNA-sequence (RNA-seq) technology, vast quantities of genomics and transcriptomics have proven to be indispensable tools that promote the advancement of precision medicine and the thorough study of numerous areas of molecular biology in autoimmune skin disorders. Furthermore, genomics and transcriptomics have been transformed by integration with 3D genomic and epigenomic data. Single-cell and spatial transcriptomic sequencing have also been developed to address intercellular transcriptomic heterogeneity at the single-cell level with coordinates. About the same time that complete genome sequences were available, two-dimensional gel electrophoresis and mass spectrometry emerged as powerful techniques for achieving the protein-identification process, which further contributed to expression proteomics and cell-map proteomics [6]. Presently, newly developed chromatographic techniques with higher resolution and sensitivity are increasingly used in proteomics, including mass spectrometry [7]. Similar to proteomics, small compounds investigated in metabolomics are separated using a chromatographic method and then sent for identification by mass spectrometry and other downstream methods such as nuclear magnetic resonance. Considering that the skin is the outer layer of the body and is inevitably colonized by microorganisms, one essential omics method for understanding inflammatory skin disease is studying the genomics of microorganisms (microbiomics), a rapidly expanding area in which all the microorganisms of a given population are researched.

In this review, we will discuss the application of genomics, transcriptomics, proteomics, and metabolomics in psoriasis and AD and how they are used and integrated for further analysis.

## 2. The Pathogenesis of Psoriasis and AD

Numerous studies have shown that the abnormal keratinocyte hyperproliferation and differentiation are driven by interactions between various immune cells and Keratinocyte. During the progression period of psoriasis, cytokines, growth factors, and antimicrobial proteins are produced and form an inflammatory circuit [8]. Keratinocytes in psoriasis patients show a higher level of oxidative stress and it is suspected that, under this stress, TNF-α and IL-6 can be secreted to activate dendric cells and promote the proliferation of IL-17-secreting cells [9,10]. As professional antigen-presenting cells, dendritic cells play a significant role in the initiation phase starting the development of the psoriatic plaque via secretion of type I IFN (IFN-α and IFN-β). Myeloid dendritic cells’ (mDC) phenotypic maturation is aided by type I IFN signaling, which has also been linked to Th1 and Th17 differentiation and function, including the generation of IFN- and interleukin IL-17, respectively. Tumor necrosis factor (TNF), IL-23, and IL-12 are secreted by activated mDCs as they move into draining lymph nodes, the latter two of which regulate the differentiation and growth of Th17 and Th1 cell subsets [11]. Furthermore, the maintenance phase of psoriatic inflammation is fueled by the adaptive immune response being activated by various T-cell subgroups [12]. In the epidermis, the Th17 cytokines IL-17, IL-21, and IL-22 stimulate keratinocyte growth. The conventional systemic medications are immunomodulators, and all but apremilast require careful clinical supervision due to their frequent side effects, which primarily affect the kidney and liver. Recent biologics differ from the systemic treatments mentioned above in that they target particular inflammatory pathways and are given subcutaneously on varying weekly schedules. The IL-23/Th17 axis and TNF signaling are currently the two pathways that biologics target as being essential for the formation and maintenance of psoriatic plaque [13].

**Figure 1:**
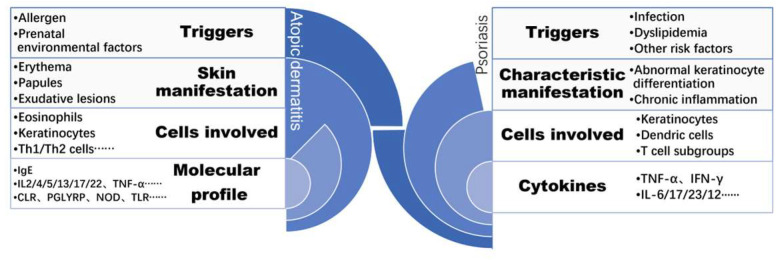
The pathogenesis of psoriasis and AD, triggers and skin manifestation of these two chronic inflammatory skin diseases differs while similar cell types and molecular profiles are involved. (PAMP (pathogen-associated molecular patterns), CLR (C-lectin receptors), PGLYRP (peptidoglycan recognition proteins), NOD (nucleotide-binding oligomerization domain), TLR (toll-like receptors), IL (interleukin), TNFα (tumor necrosis factor alpha), IFN-γ (interferon gamma), Th1 (T helper cells 1), Th1 (T helper cells 2)).

Similar to psoriasis, as shown in Figure 1, AD has a complex and multifactorial pathophysiology that includes aspects of barrier dysfunction, changes in immune responses that are cell-mediated, IgE-mediated hypersensitivity, and environmental factors. There have been genetic variations discovered that may affect the skin’s barrier function and give rise to an AD appearance [14]. The abnormalities may start with the innate immune system by an imbalance of Th2 to Th1 cytokines, which can alter cell-mediated immune responses and increase IgE-mediated hypersensitivity. The propensity for CD4 lymphocytes to differentiate into the Th2 lineage is one of the defining characteristics of this phenomenon. Toll-like receptorsTLR2 and high-affinity IgE receptor (FcRI) levels were found to be related [15]. Increased production of the cytokines IL-4, IL-5, and IL-13 is a result of excessive Th2 cell production. IgE antibodies and eosinophils are stimulated by cytokines in peripheral blood and tissues [16].

## 3. Omics Approaches Applied in Psoriasis and AD

### 3.1. Databases

In light of the open-data policy in bioinformatics, abundant resources from global investigators have been classified and gathered in various databases. One of the most important databases is the GEO database, developed and managed by NCBI, also known as Gene Expression Omnibus. The data of gene expression analyses used in the published publications can be accessed in this database, which is free to download and include high-throughput gene expression data provided by research organizations worldwide. It is crucial to note that the data may require quality control. This database has gathered a variety of omics data of many species for a number of diseases. Other databases like the Uniprot database and the Human Metabolome Database, on the other hand, place emphasis on providing molecular profiles as the results of omics studies. In Table 1, some of the most frequently used databases are listed.

**Figure 2:**
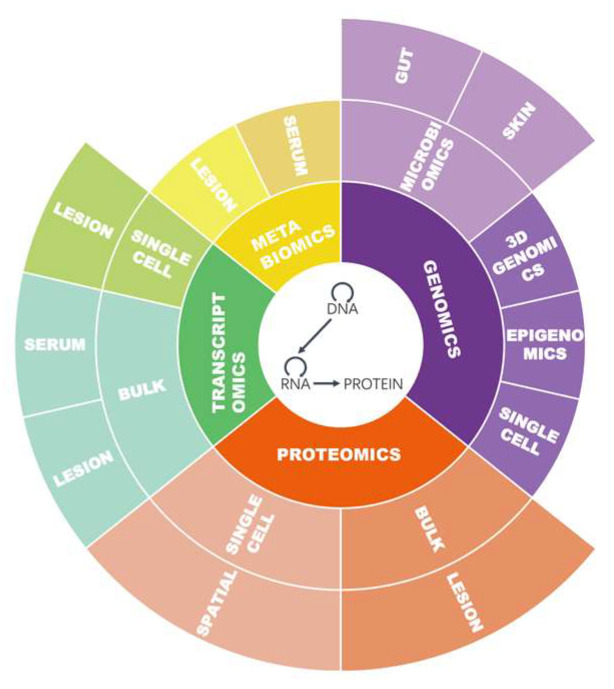
Omics data types collected on psoriasis and AD patients.

As for traditional genomics data, reported mutations in genes associated with psoriasis and AD have been analyzed countless times through the Human Gene Mutation Database [31] or other integrated databases, like MalaCards: The human disease database [32]. Up to February 24, 2023, the Gene Expression Omnibus repository of the National Library of Medicine had collected over 200 GEO dataset series and over 20,000 samples associated with psoriasis of homa sapiens (search strategy: (“psoriasis” [MeSH Terms] OR psoriasis [All Fields]) AND “Homo sapiens” [porgn]). Fewer samples were collected on AD, which include over 100 series and over 1000 samples (search strategy: (“dermatitis, atopic” [MeSH Terms] OR atopic dermatitis [All Fields]) AND “Homo sapiens” [porgn]) [33]. Due to the complexity of utilization of proteome analysis, the emphasis can be placed on multiple aspects of protein, leading to scattered omics data associated with disease. Querying the Uniprot database retrieved over 100 psoriasis-related proteins and over 30 AD-related proteins (retrieved on February 28, 2023) [34]. Databases for metabolomics like The Human Metabolome Database [35] include very few confirmed metabolic pathways and fewer than 100 metabolites with descriptions from the original article for psoriasis and AD. Even though results of microbiomics study focusing on pathogen–host interactions can be retrieved online in the Pathogen–Host Interactions (PHI) database, data of common skin disease are still lacking for the time being. In summary, as shown in Figure 2, samples from the serum and the skin lesion of patients and healthy individuals are mostly collected and prepared for extracting DNA, RNA, protein, and metabolite data. The research on etiology and the discovery of biomarkers both benefit from various omics methods.

### 3.2. Omics Approaches and Biomarkers

Each kind of omics approaches applied in psoriasis and AD often generates a list of features or biomarkers related to the disease. These biomarkers can be regarded as indicators of the diagnosis or prognosis and as clues for determining the related biological pathways or processes. To highlight the similarity of both diseases and the consistency of the molecular profiles extracted from multiple omics data, some of the shared biomarkers of psoriasis and AD are displayed in Table 2.

#### 3.2.1. Genomics

Genomics is the study of an organism’s genetic or epigenetic sequence information, which aims to comprehend the structure and function of these sequences.

##### Genome Analysis

Numerous hereditary variables are thought to cause psoriasis and AD, which can be investigated by genomics, an approach for determining how the entire genome is structured, functional, and regulated. Previously, family-based linkage disequilibrium studies discovered approximately 13 psoriasis susceptibility loci [58]. With the framework for genome-wide association studies (GWAS) of psoriasis built and single-nucleotide polymorphisms (SNPs) analyzed, the former loci have been confirmed. Genes within and beyond loci were identified such as IL12B, IL23R [59], ZNF313 [60], and TRAF31P2 [61]. These genomic biomarkers were considered potential indicators of psoriasis-causing pathways in genetically susceptible people. Besides, disparities in DNA copy number, or copy number variations (CNV), are investigated, and subsequent research revealed that CNVs in beta-defensin genes (DEFB) [62], IL22 [63], and FCGR3B [62] are associated with the risk of psoriasis.

GWAS of AD including over 21,000 cases in 2015 contributed thirty-one different chromosomal loci containing AD susceptibility genes [64]. Filaggrin, claudins, occludins-encoding genes, serine protease inhibitor gene (SPINK-5/LEKT1, cystatin A), mast cell chymase gene (CMA1), epidermal chymo-trypsin and trypsin gene, epidermal N-methyltransferase gene are all involved in the pathogenesis of AD at the level of the defective epidermal barrier [65]. Other genes that encode proteins regulating innate and acquired immune responses were investigated further in other omics study [65].

##### Epigenomics

Epigenetic studies were conducted as the first step towards understanding 3D genome structure, involving DNA demethylation, hypermethylation, hypomethylation, and histone modifications. Some methylation-sensitive genes, like SHP-1, were found in psoriasis in the initial stage [66]. Following that, global CpG methylation in psoriasis was then studied [67], and a technique known as methylated DNA immunoprecipitation sequencing (MeDIP-Seq) was developed [68]. The discovery of inverse correlations between nearby gene expression, including KYNU, OAS2, S100A12, and SERPINB3, and methylation at CpG methylation sites suggested the role of epigenetic mechanisms over important psoriatic biomarkers [66]. Patterns of DNA methylation at the PDCD5 and TIMP2 loci also provide new insights into transcriptional regulation mechanisms in psoriasis [68]. Meanwhile, an epigenome-wide association research on AD demonstrated 19 CpG sites with DNA methylation variations containing genes that are mainly concerned in keratinocyte differentiation, proliferation, and the innate immune response, S100A included [69]. Global histone H4 hypoacetylation was found in psoriatic peripheral blood mononuclear cells (PBMCs) and was negatively correlated with disease activity, as measured by the PASI score for psoriasis area severity [70]. Interactions with environmental variables were also taken into consideration, like prenatal factors. The role of prenatal exposure to smoke in shaping the epigenomic modification was confirmed by leading to hypomethylation of the TSLP 5′CpG island [71].

##### 3D Genomics

Understanding the function of chromatin topology in gene regulation is the new hotspot in the field of genetics. This involves using the 3D genomic technique to map out how chromosomes are organized and folded within the nucleus [72]. The spatial organization of chromosomes and genes in the nucleus was initially visualized by DNA fluorescence in situ hybridization (FISH), with limited genomic loci to be analyzed [73]. High-throughput single-cell sequencing techniques have been widely employed to detect variations in individual cells and changes in the genome’s structure [74]. Whole-genome and whole-exome sequencing have typically been used in single-cell research [75]. Using methods based on high-throughput chromosomal conformation capture (3C), such as high-throughput chromosome conformation capture (Hi-C), it is possible to map chromatin interactions across the whole genome [76] with or without single-cell information. Gene targets within known psoriasis GWAS loci could be annotated and linked with disease-associated enhancers, providing evidence for potential chromosome interactions in psoriasis-related cell lines [77], especially keratinocytes [78]. Only 35% of the target genes are found closest to the known GWAS variants, according to research on the interaction profile of GWAS variants during keratinocyte differentiation, and more genes have been discovered as potential novel candidates for psoriasis involvement [78]. Congeneric study in AD is still a void.

#### 3.2.2. Transcriptomics

An approach for investigating the variation in RNA levels in particular cells is called transcriptomics. It provides extensive data on mRNA and non-coding RNA profiles. Transcriptome analysis is now widely applied to discover biomarkers of psoriasis, including protein-coding genes like S100A7A [79,80,81] and miRNAs like miR-203 [82,83], which have been confirmed repeatedly in the light of the accelerated development of next-generation sequencing techniques and DNA microarray technology. Similarly, the molecular profile in AD conducted on whole skin biopsies revealed the under-expression of epidermal differentiation complex (EDC) (e.g., *FLG*, *LOC*, *S100A7/8/9*, *PI3*, *SPRR1A*, and *CLDNs*) lymphoid tissue homeostatic systems (e.g., *CCL19*, *CCL21*, and *CCR7*) [84]. MicroRNA (miRNA)-mediated mechanisms in the post-transcriptional stage were furtherly discovered in AD. MiR-155 induced by infection was discovered to be overexpressed in AD lesions, possibly leading to T-cell activation [85]. RNA-seq on biopsy specimens has been performed with single-cell [86,87,88] and spatial information [89]. Transcriptomics at the single-cell level allows for the rapid identification of cell types, such as Tc17 cell subsets and cell states [90]. Moreover, it is also possible to identify the infiltration of innate immune cells in skin lesions using algorithm CIBERSORT [91] or infer cell–cell communications mediated by ligand-receptor complexes from single-cell RNA-seq data. In AD patients, activated dendritic cells, plasma cells, resting mast cells, and CD4 naïve T cells infiltrated to an extent have shown positive correlation with CCR7 expression [92]. Such analysis also revealed decreased CCL27 in basal keratinocytes interacting with CCR10 in regulatory T cells in psoriasis [87]. While describing immune regulation, it is impossible to avoid differential pseudo-time correlation analysis, which identifies regulators of cell differentiation, transformation, and proliferation-like FOSL1 [93] in psoriasis. Spatial transcriptomics enabled two-dimensional visualization of cell clusters and provided a transcriptional profile of specific areas containing immune cells such as CD4+ and CD8+ TRM cells [89] in psoriatic plaque. It is believed that these newly developed methods will be used in AD lesions soon.

#### 3.2.3. Proteomics

As a supplement to genome translation and modification studies [6], proteomics is the approach for determining the entire proteome of tissue and cells to further identify the biomarkers for disease diagnosis and prognosis. In 2013, it started with a proteomics approach on keratome skin biopsies of psoriasis patients to discover alterations of psoriasis-related proteins S100A7, FABP5 and new potential biomarkers [94]. A large volume of proteomic data of psoriasis and AD were generated. For clinical application, proteomic signature of tape strips from AD patients was extracted to filter minimally invasive biomarkers before and after dupilumab treatment [54]. As single-cell sequencing technologies thrive and rise, single-cell proteomics has been developed and has great potential for shaping the framework of pathogenesis in psoriasis and AD.

#### 3.2.4. Metabolomics

Metabolism plays a significant role in the etiology of psoriasis and AD since it is necessary for keratinocyte inflammation, which is the shared feature of psoriasis and AD [95]. Analyzing metabolic responses to pathological and physiological stimuli in living systems using qualitative and quantitative methods is known as “metabolomics” [96]. In psoriatic lesional skin and psoriatic serum, greater concentrations of phosphatidylcholines, carnitines, and asymmetric dimethylarginine indicated enhanced cell proliferation. Unexpectedly, sphingomyelins, which suggest a poor barrier response, were not significantly increased in psoriatic skin as in AD [97]. The expression pattern of other major metabolism pathways has been investigated on psoriatic skin, focusing on the composition of skin excretions [98] and serum [99] to discover biomarkers for early diagnosis. Evidence of other metabolome profiles, like urine metabolome, are also accumulating [100]. The profile of metabolomics opens the window for studying immune–metabolism interactions in psoriasis and AD on the basis of single-cell transcriptomics and metabolomics data, which deserves to be further explored.

#### 3.2.5. Microbiomics

By amplifying and sequencing some hypervariable sections of the bacterial 16S rRNA gene, followed by grouping the sequences into operational taxonomic units, microbiomics is a new approach for analyzing the microbiome [101]. Decreased bacterial diversity was observed in psoriasis, contrary to AD, while Propionibacterium, Corynebacterium, Streptococcus, and Staphylococcus were significantly increased in psoriatic skin compared with healthy skin [102,103]. When compared to baseline or post-treatment settings, Staphylococcus sequences were more prevalent in chronic AD and linked with disease severity [104,105]. However, it was found that the severity of scalp psoriasis is correlated with a higher diversity of the scalp microbiome and the relative abundance of Pseudomonas [106]. Recent research suggests that the gut microbiome may regulate the modulatory effect on systemic immunity, which may contribute to skin barrier function [107]. It appears that psoriasis patients have decreased functional potential in the gut microbiota due to intestinal dysbiosis [108].

## 4. Multi-Omics Strategies

From the perspectives of disease etiology, diagnostic biomarkers, therapeutic targets, and treatment satisfaction, these disorders have significant unmet needs for personalized medicine. The effectiveness of treatment will be increased by using a patient’s genomic, transcriptomic, and proteomic data to diagnose diseases, anticipate drug effects and adverse effects, and select highly effective treatments and therapies. It will also help to lower medical expenses and improve the possibility that novel new pharmaceuticals will be successful in development, making it one of the industries with high expectations for the future. In addition, researchers may gain a better grasp of the flow of information starting at the underlying causes (genetic, environmental, or developmental) of disease and ending where inflammatory cascades irreversibly cause functional consequences owing to multi-omics. Therefore, multi-omics strategies are in great need to orchestrate the sequence of omics study and foster data integration. Multi-omics strategies can be classified by their initial focus as “genome first”, “phenotype first”, and “environment first” [109]. The genome- and phenotype-first approaches could be changed to the omics-first approach because of today’s abundant omics data at our fingertips, which provide more information that is not limited to genome and phenotype.

### 4.1. The Omics-First Strategy

**Figure 3:**
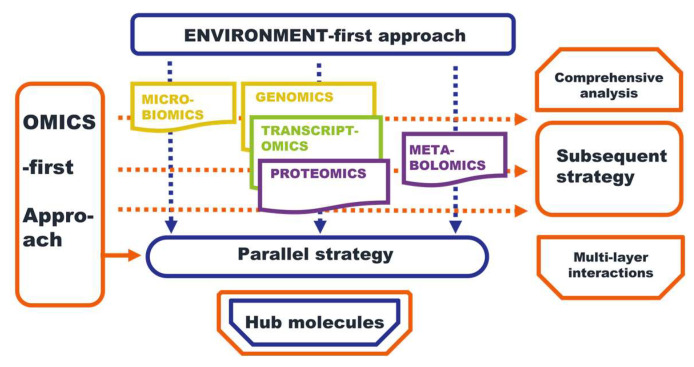
Multi-omics strategies, the blue boxes and arrows stand for the environment-first approach, while the orange boxes and arrows stand for the omics-first approach.

The omics-first strategy is based on the results of at least one omics study and usually does not rely on a complex study design. According to the correspondence of the results of different types of omics data, the omics-first strategy can be divided into parallel research and subsequent research, as shown in Figure 3.

#### 4.1.1. Parallel Research for Prioritizing Biomarkers

Parallel research is a strategy using multiple online data sources and more than one omics approach to select and confirm the reliability of a small pool of biomarkers, including proteins and protein-coding genes. For example, transcriptomics data have been repeatedly utilized to double-check the reliability of biomarkers with genomic data (CARD14) [110] or merged transcriptomic data (CCL20) by the sva package in R software (R package version 3.46.0, R version 3.2) [111]. Public transcriptomic data were integrated into a large-scale dataset to systematically annotate the gene co-expression network and reach a more persuasive conclusion [112]. More often, proteomics and transcriptomics are combined to verify the consistency of the expression of biomarkers consisting of psoriasis-related proteins and their respective coding genes. Skin proteomic studies constructed by skin taping and skin biopsy have been extensively performed to check AD biomarkers previously identified from genomics and transcriptomics (e.g., ARG1, KLK5, S100A8, FLG, and SPINKS) [113]. In the last decade, the application of parallel multi-omics research on biomarkers has expanded from diagnosis [114] to prognosis [115]. In short, the parallel research strategy applied in psoriasis and AD contributes to narrowing down the list of genes worthy of extra attention.

#### 4.1.2. Subsequent Research for Further Comprehension

Subsequent research is a strategy for excavating pathways and genes that appear insignificant when approached by a single omics research project or by investigating complex host–microorganism interactions. A small number of studies have been conducted that employ this strategy, though. The following are examples of this strategy.

##### Genomics and Transcriptomics

In order to effectively estimate gene expression and carry out transcriptome-wide association studies (TWAS), 3D genomic data can be analyzed with transcriptomics data [116] to investigate the regulation of the process of transcription. For example, the function of psoriasis-associated enhancers in the regulation of the KLF4 gene was revealed by combining the disease-focused Capture Hi-C (CHi-C) experiment, Cas9 fusion protein-mediated chromatin remodeling (CRISPR activation), and RNA-seq [77].

##### Transcriptomics and Metabolomics

Expressions of enzymes-encoding genes can be studied by transcriptomics at a low cost. Combining the transcriptomic profile of skin samples with metabolomic data in serum, the role of Lipoxygenases (LOX) and cyclooxygenase (COX) pathways for PUFA metabolism were determined in AD patients. 12/15-LOX and COX pathways were defined as upregulated pathways, while n3/n6-PUFA and metabolite ratios were lower in AD patients’ skin [117].

##### Proteomics and Metabolomics

Enzymes and metabolic reactions are inextricably linked, making them an ideal starting point for multi-omics research. Knowledge of changes in the lipidomic and proteomic profiles in psoriasis demonstrated that alterations in the lipidome are directly related to changes in the protein profile in psoriasis by examining the enzymes involved in lipidome modifications and the reaction of the endocannabinoid system to metabolic changes [118].

##### Microbiomics and Transcriptomics

Transcriptomics and microbiomics were automatically coupled in the experimental design to analyze the colonized skin and its colonizers in order to understand the skin microbe–host interplay in psoriasis. Contrary to AD, psoriasis has been shown to be characterized by co-occurring populations of microorganisms with tenuous links to the gene expression associated with the disease [119]. Gut microorganisms, which play an important role in immunity and are activated by homeostasis, have been linked to dysregulated cytokine levels in the circulatory system, including TNF-, IL-17, and IL-6 [107]. More studies concerning the crosstalk of gut microorganisms, inflammation, and immune cells are needed to clarify the role of gut microorganisms.

### 4.2. The Environment-First Approach

The environment-first approach focuses on the conditions under which the omics data are harvested. In other words, omics approaches are superior substitutes for real-time polymerase chain reaction (rt-PCR) and the Western blot method in these studies. For instance, to explore the mechanisms of unconventional medicine like the Cimicifugae Rhizoma-Smilax glabra Roxb (CS) herb pair, transcriptomics and metabolomics approaches were used to build an enzyme-gene, compound response network [120]. For investigating the mechanisms of conventional drugs, employing metabolomics on serum samples and microbiomics on stool samples before and after methotrexate (MTX) treatment provides evidence suggesting connections between the blood metabolome, gut microbiome, and effectiveness of MTX [121]. These are good examples of multi-omics approaches applied in therapeutic studies with the advantage of easily calibrated random error.

## 5. Tools for Integrating Omics Data

Integrative omics approaches currently utilized for disease study are extending and receiving a lot of attention. To divide the data into many different variations and identify differences in psoriasis patients, the majority of these integration methods first use data normalization and dimensionality reduction. Thus, mid-term multi-omics data integration describes the incorporation of various omics data types during the goal model creation procedure. In order to produce the final model results, the multi-omics data integration method primarily applies molecular data from various omics types to the target model individually. To enable the analysis and interpretation of the generated multidimensional data, the development of improved omics data integration and analysis tools is crucial. Typically, differential genes, proteins, and metabolites are generated routinely and then integrated into the gene–protein–metabolite network using the MetaboAnalyst database and the String database [122]. This method is called a multistage approach with an initial emphasis. The integration of genomics, including epigenomics, 3D genomics, and microbiomics, with other omics data shares the same workflow [123]. On the contrary, integrating multiple omics profiles in a simultaneous analysis is known as the “multi-modal approach”, which relies heavily on the analytical model such as machine learning [124]. The compendium of multi-omics datasets for another autoimmune disorder, vitiligo, has launched as an integrative database named Vitiligo Information Resource (VIRdb) [125]. For the benefit of researchers and doctors, it is hoped that a similar multi-omics database for psoriasis will be made accessible soon.

## 6. Future Perspective

The development of tools for multi-omics data integration, such as “Mergeomics” [126], and the gradual shift in the research paradigm for researchers and clinicians are currently underway. These developments result from the decreased cost of omics analyses and the increased awareness that omics studies help avoid drawing erroneous conclusions under the influence of individual differences and diverse operating skills. Without a doubt, the time has come for multi-omics strategies to help solve the disease’s jigsaw puzzle. However, challenges remain because detailed phenotype information like BMI or PASI score is missing for most omics data, which makes it difficult to extract data for further statistical analysis. Additionally, due to various data sharing policies, primary data are intermingled with downstream data in the database with or without a quality control report, increasing the difficulty of reutilization of online data and undermining the credibility of multi-omics research.

With a more advanced integrated omics approach, systems biology is gaining popularity. In order to better identify information with diagnostic, prognostic, or therapeutic potential, this strategy is utilized to examine the connections between various molecular levels [127]. For a thorough examination of molecular data, bioinformatics and clinical expertise must be combined to offer a full perspective on the disorders. Only a few clinical practice studies on systems biology are now available in psoriasis and AD, and more studies are required.

Nonetheless, multi-omics has already altered the perception of psoriasis early diagnosis and prognosis, as well as interpreted psoriasis pathogenesis in novel ways. Medical research in the future will focus on preventive measures that fit into our daily lives, individualized therapies, and future surveillance of each person’s health indicators. Despite the considerable gap between research discovery and clinical application, multi-omics research has great potential for revolutionizing the processes of risk evaluation, diagnosis, personalized treatment plan, and prognosis for psoriasis patients and the susceptible population.

## Figures and Tables

**Table 1:** Commonly used databases of omics data.

Database Name	Database Full Name	Application	Ref.
GEO	Gene Expression Omnibus repository of the National Library of Medicine	All	[17]
HGMD	Human Gene Mutation Database	Genomics	[18]
Ensembl	Ensembl	Genomics	[19]
ClinVar	ClinVar	Genomics	[20]
eFORGE	the epigenetic equivalent of FORGE, using EWAS rather than GWAS data	Epigenomics	[21]
HMDD	the Human microRNA Disease Database	MicroRNA	[22]
circRNADisease	circRNADisease	CircRNA	[23]
lncRNADisease	lncRNADisease	lncRNA	[24]
Uniprot	Uniprot	Proteomics	[25]
SMART	Simple Modular Architecture Research Tool	Proteomics	[26]
PTMD	Post-Translational Modification Database	Proteomics	[27]
HMDB	The Human Metabolome Database	Metabiomics	[28]
PHI	Pathogen–Host Interactions Database	Microbiomics	[29]
HMP	Human Microbiome Project Data Portal	Microbiomics	[30]

**Table 2:** Common omics markers of psoriasis and AD.

Pathways	Marker Name	Omics Type	Disease	Ref.
Epidermal differentiation pathway	S100A8(SNP)	genomics	Psoriasis	[36]
	S100A8	transcriptomics	Psoriasis	[37]
	S100A8	transcriptomics	AD	[38]
	S100A8(protein)	proteomics	AD	[39]
	S100A8(protein)	proteomics	Psoriasis	[40]
IL-17-mediated inflammatory Pathway	IL-1a(SNP)	genomics	AD	[41]
	IL-1a	transcriptomics	Psoriasis	[42]
IL-31 Pathway	IL-31	transcriptomics	Psoriasis	[43]
	IL-31	transcriptomics	AD	[44]
Calcium-permeable cation TRPs channels	TRPV1(SNP)	genomics	AD	[45]
	TRPV1	transcriptomics	Psoriasis	[46]
	TRPV1	transcriptomics	AD	[44]
TH1 cytokine	IL-13(SNP)	genomics	AD	[46]
	IL-13	genomics	Psoriasis	[47]
	IL-13	transcriptomics	Psoriasis	[43]
	IL-13	transcriptomics	AD	[48]
	IL-13	transcriptomics	AD	[49]
	IL-13(protein)	proteomics	AD	[50]
IL-20 family cytokine	IL-24(haplotype)	genomics	Psoriasis	[51]
	IL-24	transcriptomics	Psoriasis	[43]
	IL-24	transcriptomics	AD	[52]
Immunomodulatory chemokines	CXCL1	transcriptomics	Psoriasis	[42]
	CXCL1	transcriptomics	AD	[38]
	CXCL1	proteomics	Psoriasis	[53]
	CXCL1	proteomics	AD	[54]
IL-1 pathways	IL-36(SNP)	genomics	psoriasis	[55]
	IL-36	transcriptomics	Psoriasis	[56]
	IL-36	transcriptomics	AD	[57]

## Data Availability

Not applicable.

## References

[1] Griffiths C.E.M., Armstrong A.W., Gudjonsson J.E., Barker J.N.W.N. (2021). Psoriasis. Lancet.

[2] Ständer S. (2021). Atopic Dermatitis. N. Engl. J. Med..

[3] Griffiths C.E., Barker J.N. (2007). Pathogenesis and clinical features of psoriasis. Lancet.

[4] Nickoloff B.J. (2007). Cracking the cytokine code in psoriasis. Nat. Med..

[5] Crick F. (1970). Central dogma of molecular biology. Nature.

[6] Blackstock W.P., Weir M.P. (1999). Proteomics: Quantitative and physical mapping of cellular proteins. Trends Biotechnol..

[7] Venkatesh A., Patel S.K., Ray S., Shastri J., Chatterjee G., Kochar S.K., Patankar S., Srivastava S. (2016). Proteomics of Plasmodium vivax malaria: New insights, progress and potential. Expert Rev. Proteom..

[8] Kennedy-Crispin M., Billick E., Mitsui H., Gulati N., Fujita H., Gilleaudeau P., Sullivan-Whalen M., Johnson-Huang L.M., Suárez-Fariñas M., Krueger J.G. (2012). Human keratinocytes’ response to injury upregulates CCL20 and other genes linking innate and adaptive immunity. J. Investig. Dermatol..

[9] Buckley L.H., Xiao R., Perman M.J., Grossman A.B., Weiss P.F. (2021). Psoriasis Associated With Tumor Necrosis Factor Inhibitors in Children With Inflammatory Diseases. Arthritis Care Res..

[10] Renne J., Schäfer V., Werfel T., Wittmann M. (2010). Interleukin-1 from epithelial cells fosters T cell-dependent skin inflammation. Br. J. Dermatol..

[11] Hänsel A., Günther C., Ingwersen J., Starke J., Schmitz M., Bachmann M., Meurer M., Rieber E.P., Schäkel K. (2011). Human slan (6-sulfo LacNAc) dendritic cells are inflammatory dermal dendritic cells in psoriasis and drive strong TH17/TH1 T-cell responses. J. Allergy Clin. Immunol..

[12] Nestle F.O., Turka L.A., Nickoloff B.J. (1994). Characterization of dermal dendritic cells in psoriasis. Autostimulation of T lymphocytes and induction of Th1 type cytokines. J. Clin. Investig..

[13] Rendon A., Schäkel K. (2019). Psoriasis Pathogenesis and Treatment. Int. J. Mol. Sci..

[14] David Boothe W., Tarbox J.A., Tarbox M.B. (2017). Atopic Dermatitis: Pathophysiology. Adv. Exp. Med. Biol..

[15] Sroka-Tomaszewska J., Trzeciak M. (2021). Molecular Mechanisms of Atopic Dermatitis Pathogenesis. Int. J. Mol. Sci..

[16] Matsunaga M.C., Yamauchi P.S. (2016). IL-4 and IL-13 Inhibition in Atopic Dermatitis. J. Drugs Dermatol..

[17] Gene Expression Omnibus Repository of National Library of Medicine. https://www.ncbi.nlm.nih.gov/.

[18] Human Gene Mutation Database. https://www.hgmd.cf.ac.uk/ac/index.php.

[19] Ensembl. http://ensembl.org/index.html.

[20] ClinVar. https://www.ncbi.nlm.nih.gov/clinvar/.

[21] The Epigenetic Equivalent of FORGE, Using EWAS Rather than GWAS Data. https://eforge.altiusinstitute.org/.

[22] The Human microRNA Disease Database. http://www.cuilab.cn/hmdd.

[23] circRNADisease. http://cgga.org.cn:9091/circRNADisease/.

[24] lncRNADisease. http://www.rnanut.net/lncrnadisease/.

[25] Uniprot. https://www.uniprot.org/.

[26] Simple Modular Architecture Research Tool. http://smart.embl-heidelberg.de/.

[27] Post Translational Modification Database. modificationdata-basehttp://ptmd.biocuckoo.org/.

[28] The Human Metabolome Database. https://hmdb.ca/.

[29] Pathogen Host Interactions Database. http://www.phi-base.org/.

[30] Human Microbiome Project Data Portal. https://portal.hmpdacc.org/.

[31] Stenson P.D., Ball E.V., Mort M., Phillips A.D., Shiel J.A., Thomas N.S.T., Abeysinghe S., Krawczak M., Cooper D.N. (2003). Human Gene Mutation Database (HGMD): 2003 update. Hum. Mutat..

[32] Rappaport N., Twik M., Plaschkes I., Nudel R., Iny Stein T., Levitt J., Gershoni M., Morrey C.P., Safran M., Lancet D. (2017). MalaCards: An amalgamated human disease compendium with diverse clinical and genetic annotation and structured search. Nucleic Acids Res..

[33] National Center for Biotechnology Information (NCBI) [Internet] Bethesda (MD): National Library of Medicine (US), National Center for Biotechnology Information. https://www.ncbi.nlm.nih.gov/.

[34] The UniProt Consortium UniProt: The Universal Protein Knowledgebase in 2023. https://www.uniprot.org/uniprotkb?query=psoriasis.

[35] Wishart D.S., Guo A., Oler E., Wang F., Anjum A., Peters H., Dizon R., Sayeeda Z., Tian S., Lee B.L. (2022). HMDB 5.0: The Human Metabolome Database for 2022. Nucleic Acids Res..

[36] Farag A., Shoaib M., Labeeb A., Sleem A., Hussien H., Elshaib M., Hanout H. (2022). S100A8 (rs3806232) gene polymorphism and S100A8 serum level in psoriasis vulgaris patients: A preliminary study. J. Cosmet. Dermatol..

[37] Wang L., Yu X., Wu C., Zhu T., Wang W., Zheng X., Jin H. (2018). RNA sequencing-based longitudinal transcriptomic profiling gives novel insights into the disease mechanism of generalized pustular psoriasis. BMC Med. Genom..

[38] Tsoi L.C., Rodriguez E., Stölzl D., Wehkamp U., Sun J., Gerdes S., Sarkar M.K., Hübenthal M., Zeng C., Uppala R. (2020). Progression of acute-to-chronic atopic dermatitis is associated with quantitative rather than qualitative changes in cytokine responses. J. Allergy Clin. Immunol..

[39] Gittler J.K., Shemer A., Suárez-Fariñas M., Fuentes-Duculan J., Gulewicz K.J., Wang C.Q.F., Mitsui H., Cardinale I., de Guzman Strong C., Krueger J.G. (2012). Progressive activation of T(H)2/T(H)22 cytokines and selective epidermal proteins characterizes acute and chronic atopic dermatitis. J. Allergy Clin. Immunol..

[40] Schonthaler H.B., Guinea-Viniegra J., Wculek S.K., Ruppen I., Ximénez-Embún P., Guío-Carrión A., Navarro R., Hogg N., Ashman K., Wagner E.F. (2013). S100A8-S100A9 protein complex mediates psoriasis by regulating the expression of complement factor C3. Immunity.

[41] Babić Ž., Sabolić Pipinić I., Varnai V.M., Kežić S., Macan J. (2016). Associations of TNFα-308G>A, TNFα-238G>A, IL-1α-889C>T and IL-10 -1082G>A Genetic Polymorphisms with Atopic Diseases: Asthma, Rhinitis and Dermatitis. Int. Arch. Allergy Immunol..

[42] Lowes M.A., Suárez-Fariñas M., Krueger J.G. (2014). Immunology of psoriasis. Annu. Rev. Immunol..

[43] Pasquali L., Srivastava A., Meisgen F., Das Mahapatra K., Xia P., Xu Landén N., Pivarcsi A., Sonkoly E. (2019). The Keratinocyte Transcriptome in Psoriasis: Pathways Related to Immune Responses, Cell Cycle and Keratinization. Acta Derm. Venereol..

[44] Möbus L., Rodriguez E., Harder I., Stölzl D., Boraczynski N., Gerdes S., Kleinheinz A., Abraham S., Heratizadeh A., Handrick C. (2021). Atopic dermatitis displays stable and dynamic skin transcriptome signatures. J. Allergy Clin. Immunol..

[45] Rodrigues de Souza I., Savio de Araujo-Souza P., Morais Leme D. (2022). Genetic variants affecting chemical mediated skin immunotoxicity. J. Toxicol. Environ. Health Part B Crit. Rev..

[46] Hirota T., Takahashi A., Kubo M., Tsunoda T., Tomita K., Sakashita M., Yamada T., Fujieda S., Tanaka S., Doi S. (2012). Genome-wide association study identifies eight new susceptibility loci for atopic dermatitis in the Japanese population. Nat. Genet..

[47] Nanda H., Ponnusamy N., Odumpatta R., Jeyakanthan J., Mohanapriya A. (2020). Exploring genetic targets of psoriasis using genome wide association studies (GWAS) for drug repurposing. 3 Biotech.

[48] Oetjen L.K., Mack M.R., Feng J., Whelan T.M., Niu H., Guo C.J., Chen S., Trier A.M., Xu A.Z., Tripathi S.V. (2017). Sensory Neurons Co-opt Classical Immune Signaling Pathways to Mediate Chronic Itch. Cell.

[49] Beck L.A., Thaçi D., Hamilton J.D., Graham N.M., Bieber T., Rocklin R., Ming J.E., Ren H., Kao R., Simpson E. (2014). Dupilumab treatment in adults with moderate-to-severe atopic dermatitis. N. Engl. J. Med..

[50] Brunner P.M., Suárez-Fariñas M., He H., Malik K., Wen H.-C., Gonzalez J., Chan T.C.-C., Estrada Y., Zheng X., Khattri S. (2017). The atopic dermatitis blood signature is characterized by increases in inflammatory and cardiovascular risk proteins. Sci. Rep..

[51] Kõks S., Kingo K., Vabrit K., Rätsep R., Karelson M., Silm H., Vasar E. (2005). Possible relations between the polymorphisms of the cytokines IL-19, IL-20 and IL-24 and plaque-type psoriasis. Genes Immun..

[52] Vu Y.H., Hashimoto-Hachiya A., Takemura M., Yumine A., Mitamura Y., Nakahara T., Furue M., Tsuji G. (2020). IL-24 Negatively Regulates Keratinocyte Differentiation Induced by Tapinarof, an Aryl Hydrocarbon Receptor Modulator: Implication in the Treatment of Atopic Dermatitis. Int. J. Mol. Sci..

[53] Méhul B., Laffet G., Séraïdaris A., Russo L., Fogel P., Carlavan I., Pernin C., Andres P., Queille-Roussel C., Voegel J.J. (2017). Noninvasive proteome analysis of psoriatic stratum corneum reflects pathophysiological pathways and is useful for drug profiling. Br. J. Dermatol..

[54] He H., Olesen C.M., Pavel A.B., Clausen M.-L., Wu J., Estrada Y., Zhang N., Agner T., Guttman-Yassky E. (2020). Tape-Strip Proteomic Profiling of Atopic Dermatitis on Dupilumab Identifies Minimally Invasive Biomarkers. Front. Immunol..

[55] Traks T., Keermann M., Prans E., Karelson M., Loite U., Kõks G., Silm H., Kõks S., Kingo K. (2019). Polymorphisms in IL36G gene are associated with plaque psoriasis. BMC Med. Genet..

[56] Chiricozzi A., Guttman-Yassky E., Suárez-Fariñas M., Nograles K.E., Tian S., Cardinale I., Chimenti S., Krueger J.G. (2011). Integrative responses to IL-17 and TNF-α in human keratinocytes account for key inflammatory pathogenic circuits in psoriasis. J. Investig. Dermatol..

[57] Suárez-Fariñas M., Ungar B., Correa da Rosa J., Ewald D.A., Rozenblit M., Gonzalez J., Xu H., Zheng X., Peng X., Estrada Y.D. (2015). RNA sequencing atopic dermatitis transcriptome profiling provides insights into novel disease mechanisms with potential therapeutic implications. J. Allergy Clin. Immunol..

[58] Puig L., Julià A., Marsal S. (2014). The pathogenesis and genetics of psoriasis. Actas Dermo Sifiliográficas.

[59] Capon F., Di Meglio P., Szaub J., Prescott N.J., Dunster C., Baumber L., Timms K., Gutin A., Abkevic V., Burden A.D. (2007). Sequence variants in the genes for the interleukin-23 receptor (IL23R) and its ligand (IL12B) confer protection against psoriasis. Hum. Genet..

[60] Capon F., Bijlmakers M.-J., Wolf N., Quaranta M., Huffmeier U., Allen M., Timms K., Abkevich V., Gutin A., Smith R. (2008). Identification of ZNF313/RNF114 as a novel psoriasis susceptibility gene. Hum. Mol. Genet..

[61] Hüffmeier U., Uebe S., Ekici A.B., Bowes J., Giardina E., Korendowych E., Juneblad K., Apel M., McManus R., Ho P. (2010). Common variants at TRAF3IP2 are associated with susceptibility to psoriatic arthritis and psoriasis. Nat. Genet..

[62] Wu Y., Zhang Z., Tao L., Chen G., Liu F., Wang T., Xue F., Chen Y., He L., Zheng J. (2014). A high copy number of FCGR3B is associated with psoriasis vulgaris in Han Chinese. Dermatology.

[63] Prans E., Kingo K., Traks T., Silm H., Vasar E., Kõks S. (2013). Copy number variations in IL22 gene are associated with Psoriasis vulgaris. Hum. Immunol..

[64] Paternoster L., Standl M., Waage J., Baurecht H., Hotze M., Strachan D.P., Curtin J.A., Bønnelykke K., Tian C., Takahashi A. (2015). Multi-ancestry genome-wide association study of 21,000 cases and 95,000 controls identifies new risk loci for atopic dermatitis. Nat. Genet..

[65] Nedoszytko B., Reszka E., Gutowska-Owsiak D., Trzeciak M., Lange M., Jarczak J., Niedoszytko M., Jablonska E., Romantowski J., Strapagiel D. (2020). Genetic and Epigenetic Aspects of Atopic Dermatitis. Int. J. Mol. Sci..

[66] Ruchusatsawat K., Wongpiyabovorn J., Shuangshoti S., Hirankarn N., Mutirangura A. (2006). SHP-1 promoter 2 methylation in normal epithelial tissues and demethylation in psoriasis. J. Mol. Med..

[67] Roberson E.D.O., Liu Y., Ryan C., Joyce C.E., Duan S., Cao L., Martin A., Liao W., Menter A., Bowcock A.M. (2012). A subset of methylated CpG sites differentiate psoriatic from normal skin. J. Investig. Dermatol..

[68] Zhang P., Zhao M., Liang G., Yin G., Huang D., Su F., Zhai H., Wang L., Su Y., Lu Q. (2013). Whole-genome DNA methylation in skin lesions from patients with psoriasis vulgaris. J. Autoimmun..

[69] Rodríguez E., Baurecht H., Wahn A.F., Kretschmer A., Hotze M., Zeilinger S., Klopp N., Illig T., Schramm K., Prokisch H. (2014). An integrated epigenetic and transcriptomic analysis reveals distinct tissue-specific patterns of DNA methylation associated with atopic dermatitis. J. Investig. Dermatol..

[70] Zhang P., Su Y., Zhao M., Huang W., Lu Q. (2011). Abnormal histone modifications in PBMCs from patients with psoriasis vulgaris. Eur. J. Dermatol..

[71] Mu Z., Zhang J. (2020). The Role of Genetics, the Environment, and Epigenetics in Atopic Dermatitis. Adv. Exp. Med. Biol..

[72] Kempfer R., Pombo A. (2020). Methods for mapping 3D chromosome architecture. Nat. Rev. Genet..

[73] Croft J.A., Bridger J.M., Boyle S., Perry P., Teague P., Bickmore W.A. (1999). Differences in the localization and morphology of chromosomes in the human nucleus. J. Cell Biol..

[74] Yasen A., Aini A., Wang H., Li W., Zhang C., Ran B., Tuxun T., Maimaitinijiati Y., Shao Y., Aji T. (2020). Progress and applications of single-cell sequencing techniques. Infect. Genet. Evol..

[75] Gawad C., Koh W., Quake S.R. (2016). Single-cell genome sequencing: Current state of the science. Nat. Rev. Genet..

[76] Rao S.S.P., Huntley M.H., Durand N.C., Stamenova E.K., Bochkov I.D., Robinson J.T., Sanborn A.L., Machol I., Omer A.D., Lander E.S. (2014). A 3D map of the human genome at kilobase resolution reveals principles of chromatin looping. Cell.

[77] Ray-Jones H., Duffus K., McGovern A., Martin P., Shi C., Hankinson J., Gough O., Yarwood A., Morris A.P., Adamson A. (2020). Mapping DNA interaction landscapes in psoriasis susceptibility loci highlights KLF4 as a target gene in 9q31. BMC Biol..

[78] Sahlén P., Spalinskas R., Asad S., Mahapatra K.D., Höjer P., Anil A., Eisfeldt J., Srivastava A., Nikamo P., Mukherjee A. (2021). Chromatin interactions in differentiating keratinocytes reveal novel atopic dermatitis- and psoriasis-associated genes. J Allergy Clin. Immunol..

[79] Gudjonsson J.E., Ding J., Johnston A., Tejasvi T., Guzman A.M., Nair R.P., Voorhees J.J., Abecasis G.R., Elder J.T. (2010). Assessment of the psoriatic transcriptome in a large sample: Additional regulated genes and comparisons with in vitro models. J. Investig. Dermatol..

[80] Keermann M., Kõks S., Reimann E., Prans E., Abram K., Kingo K. (2015). Transcriptional landscape of psoriasis identifies the involvement of IL36 and IL36RN. BMC Genom..

[81] Oestreicher J.L., Walters I.B., Kikuchi T., Gilleaudeau P., Surette J., Schwertschlag U., Dorner A.J., Krueger J.G., Trepicchio W.L. (2001). Molecular classification of psoriasis disease-associated genes through pharmacogenomic expression profiling. The Pharm. J..

[82] Xu Y., Ji Y., Lan X., Gao X., Chen H.-D., Geng L. (2017). miR-203 contributes to IL-17-induced VEGF secretion by targeting SOCS3 in keratinocytes. Mol. Med. Rep..

[83] Joyce C.E., Zhou X., Xia J., Ryan C., Thrash B., Menter A., Zhang W., Bowcock A.M. (2011). Deep sequencing of small RNAs from human skin reveals major alterations in the psoriasis miRNAome. Hum. Mol. Genet..

[84] Schwingen J., Kaplan M., Kurschus F.C. (2020). Review-Current Concepts in Inflammatory Skin Diseases Evolved by Transcriptome Analysis: In-Depth Analysis of Atopic Dermatitis and Psoriasis. Int. J. Mol. Sci..

[85] Quinn S.R., Mangan N.E., Caffrey B.E., Gantier M.P., Williams B.R.G., Hertzog P.J., McCoy C.E., O’Neill L.A.J. (2014). The role of Ets2 transcription factor in the induction of microRNA-155 (miR-155) by lipopolysaccharide and its targeting by interleukin-10. J. Biol. Chem..

[86] Nakamizo S., Dutertre C.-A., Khalilnezhad A., Zhang X.M., Lim S., Lum J., Koh G., Foong C., Yong P.J.A., Tan K.J. (2021). Single-cell analysis of human skin identifies CD14^+^ type 3 dendritic cells co-producing IL1B and IL23A in psoriasis. J. Exp. Med..

[87] Kim J., Lee J., Kim H.J., Kameyama N., Nazarian R., Der E., Cohen S., Guttman-Yassky E., Putterman C., Krueger J.G. (2021). Single-cell transcriptomics applied to emigrating cells from psoriasis elucidate pathogenic versus regulatory immune cell subsets. J. Allergy Clin. Immunol..

[88] Qie C., Jiang J., Liu W., Hu X., Chen W., Xie X., Liu J. (2020). Single-cell RNA-Seq reveals the transcriptional landscape and heterogeneity of skin macrophages in Vsir^-/-^ murine psoriasis. Theranostics.

[89] Reschke R., Shapiro J.W., Yu J., Rouhani S.J., Olson D.J., Zha Y., Gajewski T.F. (2022). Checkpoint Blockade-Induced Dermatitis and Colitis Are Dominated by Tissue-Resident Memory T Cells and Th1/Tc1 Cytokines. Cancer Immunol. Res..

[90] Huang T., Gao Z., Zhang Y., Fan K., Wang F., Li Y., Zhong J., Fan H.Y., Cao Q., Zhou J. (2018). CRL4^DCAF2^ negatively regulates IL-23 production in dendritic cells and limits the development of psoriasis. J. Exp. Med..

[91] Gong X., Wang W. (2021). Profiles of Innate Immune Cell Infiltration and Related Core Genes in Psoriasis. BioMed Res. Int..

[92] Li C., Lu Y., Han X. (2022). Identification of Effective Diagnostic Biomarkers and Immune Cell Infiltration in Atopic Dermatitis by Comprehensive Bioinformatics Analysis. Front. Mol. Biosci..

[93] Zeng F., Liu H., Lu D., Liu Q., Chen H., Zheng F. (2019). Integrated analysis of gene expression profiles identifies transcription factors potentially involved in psoriasis pathogenesis. J. Cell. Biochem..

[94] Williamson J.C., Scheipers P., Schwämmle V., Zibert J.R., Beck H.C., Jensen O.N. (2013). A proteomics approach to the identification of biomarkers for psoriasis utilising keratome biopsy. J. Proteom..

[95] Zhou X., Chen Y., Cui L., Shi Y., Guo C. (2022). Advances in the pathogenesis of psoriasis: From keratinocyte perspective. Cell Death Dis..

[96] Nicholson J.K., Lindon J.C., Holmes E. (1999). ‘Metabonomics’: Understanding the metabolic responses of living systems to pathophysiological stimuli via multivariate statistical analysis of biological NMR spectroscopic data. Xenobiotica.

[97] Ilves L., Ottas A., Kaldvee B., Abram K., Soomets U., Zilmer M., Jaks V., Kingo K. (2022). Metabolomic Differences between the Skin and Blood Sera of Atopic Dermatitis and Psoriasis. Int. J. Mol. Sci..

[98] Dutkiewicz E.P., Hsieh K.-T., Wang Y.-S., Chiu H.-Y., Urban P.L. (2016). Hydrogel Micropatch and Mass Spectrometry-Assisted Screening for Psoriasis-Related Skin Metabolites. Clin. Chem..

[99] Kang H., Li X., Zhou Q., Quan C., Xue F., Zheng J., Yu Y. (2017). Exploration of candidate biomarkers for human psoriasis based on gas chromatography-mass spectrometry serum metabolomics. Br. J. Dermatol..

[100] Alonso A., Julià A., Vinaixa M., Domènech E., Fernández-Nebro A., Cañete J.D., Ferrándiz C., Tornero J., Gisbert J.P., Nos P. (2016). Urine metabolome profiling of immune-mediated inflammatory diseases. BMC Med..

[101] Klindworth A., Pruesse E., Schweer T., Peplies J., Quast C., Horn M., Glöckner F.O. (2013). Evaluation of general 16S ribosomal RNA gene PCR primers for classical and next-generation sequencing-based diversity studies. Nucleic Acids Res..

[102] Alekseyenko A.V., Perez-Perez G.I., De Souza A., Strober B., Gao Z., Bihan M., Li K., Methé B.A., Blaser M.J. (2013). Community differentiation of the cutaneous microbiota in psoriasis. Microbiome.

[103] Gao Z., Tseng C.-h., Strober B.E., Pei Z., Blaser M.J. (2008). Substantial alterations of the cutaneous bacterial biota in psoriatic lesions. PLoS ONE.

[104] Kong H.H., Oh J., Deming C., Conlan S., Grice E.A., Beatson M.A., Nomicos E., Polley E.C., Komarow H.D., Murray P.R. (2012). Temporal shifts in the skin microbiome associated with disease flares and treatment in children with atopic dermatitis. Genome Res..

[105] Chng K.R., Tay A.S.L., Li C., Ng A.H.Q., Wang J., Suri B.K., Matta S.A., McGovern N., Janela B., Wong X.F.C.C. (2016). Whole metagenome profiling reveals skin microbiome-dependent susceptibility to atopic dermatitis flare. Nat. Microbiol..

[106] Choi J.-Y., Kim H., Koo H.-Y.-R., You J., Yu D.-S., Lee Y.-B., Lee M. (2022). Severe Scalp Psoriasis Microbiome Has Increased Biodiversity and Relative Abundance of Pseudomonas Compared to Mild Scalp Psoriasis. J. Clin. Med..

[107] Zhang X., Shi L., Sun T., Guo K., Geng S. (2021). Dysbiosis of gut microbiota and its correlation with dysregulation of cytokines in psoriasis patients. BMC Microbiol..

[108] Todberg T., Egeberg A., Zachariae C., Sørensen N., Pedersen O., Skov L. (2022). Patients with psoriasis have a dysbiotic taxonomic and functional gut microbiota. Br. J. Dermatol..

[109] Hasin Y., Seldin M., Lusis A. (2017). Multi-omics approaches to disease. Genome Biol..

[110] Harden J.L., Lewis S.M., Pierson K.C., Suárez-Fariñas M., Lentini T., Ortenzio F.S., Zaba L.C., Goldbach-Mansky R., Bowcock A.M., Lowes M.A. (2014). CARD14 expression in dermal endothelial cells in psoriasis. PLoS ONE.

[111] Elnabawi Y.A., Garshick M.S., Tawil M., Barrett T.J., Fisher E.A., Lo Sicco K., Neimann A.L., Scher J.U., Krueger J., Berger J.S. (2021). CCL20 in psoriasis: A potential biomarker of disease severity, inflammation, and impaired vascular health. J. Am. Acad. Dermatol..

[112] Federico A., Pavel A., Möbus L., McKean D., Del Giudice G., Fortino V., Niehues H., Rastrick J., Eyerich K., Eyerich S. (2022). The integration of large-scale public data and network analysis uncovers molecular characteristics of psoriasis. Hum. Genom..

[113] Ghosh D., Bernstein J.A., Khurana Hershey G.K., Rothenberg M.E., Mersha T.B. (2018). Leveraging Multilayered "Omics" Data for Atopic Dermatitis: A Road Map to Precision Medicine. Front. Immunol..

[114] Piruzian E., Bruskin S., Ishkin A., Abdeev R., Moshkovskii S., Melnik S., Nikolsky Y., Nikolskaya T. (2010). Integrated network analysis of transcriptomic and proteomic data in psoriasis. BMC Syst. Biol..

[115] Wang X., Kaiser H., Kvist-Hansen A., McCauley B.D., Skov L., Hansen P.R., Becker C. (2022). IL-17 Pathway Members as Potential Biomarkers of Effective Systemic Treatment and Cardiovascular Disease in Patients with Moderate-to-Severe Psoriasis. Int. J. Mol. Sci..

[116] Khunsriraksakul C., McGuire D., Sauteraud R., Chen F., Yang L., Wang L., Hughey J., Eckert S., Dylan Weissenkampen J., Shenoy G. (2022). Integrating 3D genomic and epigenomic data to enhance target gene discovery and drug repurposing in transcriptome-wide association studies. Nat. Commun..

[117] Töröcsik D., Weise C., Gericke J., Szegedi A., Lucas R., Mihaly J., Worm M., Rühl R. (2019). Transcriptomic and lipidomic profiling of eicosanoid/docosanoid signalling in affected and non-affected skin of human atopic dermatitis patients. Exp. Dermatol..

[118] Łuczaj W., Gęgotek A., Skrzydlewska E. (2021). Analytical approaches to assess metabolic changes in psoriasis. J. Pharm. Biomed. Anal..

[119] Fyhrquist N., Muirhead G., Prast-Nielsen S., Jeanmougin M., Olah P., Skoog T., Jules-Clement G., Feld M., Barrientos-Somarribas M., Sinkko H. (2019). Microbe-host interplay in atopic dermatitis and psoriasis. Nat. Commun..

[120] Hu X., Qi C., Feng F., Wang Y., Di T., Meng Y., Wang Y., Zhao N., Zhang X., Li P. (2022). Combining network pharmacology, RNA-seq, and metabolomics strategies to reveal the mechanism of Cimicifugae Rhizoma—Smilax glabra Roxb herb pair for the treatment of psoriasis. Phytomedicine.

[121] Qiu Q., Deng J., Deng H., Yao D., Yan Y., Ye S., Shang X., Deng Y., Han L., Zheng G. (2022). Association of the characteristics of the blood metabolome and gut microbiome with the outcome of methotrexate therapy in psoriasis. Front. Immunol..

[122] Yang G., Zhou S., He H., Shen Z., Liu Y., Hu J., Wang J. (2022). Exploring the “gene-protein-metabolite” network of coronary heart disease with phlegm and blood stasis syndrome by integrated multi-omics strategy. Front. Immunol..

[123] Zhao Y., Jhamb D., Shu L., Arneson D., Rajpal D.K., Yang X. (2019). Multi-omics integration reveals molecular networks and regulators of psoriasis. BMC Syst. Biol..

[124] Agamah F.E., Bayjanov J.R., Niehues A., Njoku K.F., Skelton M., Mazandu G.K., Ederveen T.H.A., Mulder N., Chimusa E.R., ‘t Hoen P.A.C. (2022). Computational approaches for network-based integrative multi-omics analysis. Front. Mol. Biosci..

[125] Srivastava P., Talwar M., Yadav A., Choudhary A., Mohanty S., Bharti S., Narad P., Sengupta A. (2020). VIRdb 2.0: Interactive analysis of comorbidity conditions associated with vitiligo pathogenesis using co-expression network-based approach. F1000Research.

[126] Ding J., Blencowe M., Nghiem T., Ha S.-M., Chen Y.-W., Li G., Yang X. (2021). Mergeomics 2.0: A web server for multi-omics data integration to elucidate disease networks and predict therapeutics. Nucleic Acids Res..

[127] Cisek K., Krochmal M., Klein J., Mischak H. (2016). The application of multi-omics and systems biology to identify therapeutic targets in chronic kidney disease. Nephrol. Dial. Transplant..

